# (*E*)-1-Phenyl-3-[4-(trifluoro­meth­yl)phen­yl]prop-2-en-1-one

**DOI:** 10.1107/S1600536812035763

**Published:** 2012-08-23

**Authors:** Pei-Hua Zhao, Er-Jun Hao, Ya-Qing Liu, Gui-Zhe Zhao

**Affiliations:** a Research Center for Engineering Technology of Polymeric Composites of Shanxi Province, College of Materials Science and Engineering, North University of China, Taiyuan 030051, People’s Republic of China; b Key Laboratory of Green Chemical Media and Reactions, Ministry of Education, College of Chemistry and Environmental Science, Henan Normal University, Xinxiang 453007, People’s Republic of China

## Abstract

In the title compound, C_16_H_11_F_3_O, the dihedral angle between the two rings is 48.8 (2)°. The crystal packing exhibits no classical inter­molecular inter­actions between the mol­ecules.

## Related literature

For applications of related compounds, see: Shibata (1994 ▶); Devincenzo *et al.* (1995 ▶); Dimmock *et al.* (1999 ▶); Go *et al.* (2005 ▶).
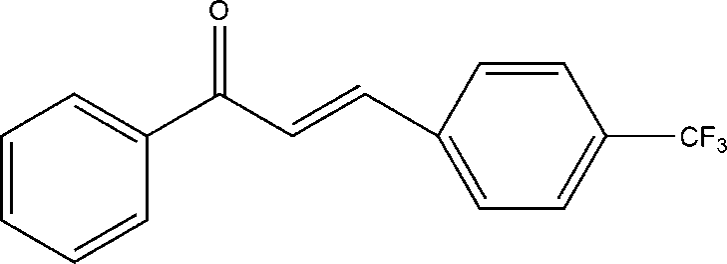


## Experimental

### 

#### Crystal data

C_16_H_11_F_3_O
*M* *_r_* = 276.25
Monoclinic, 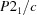

*a* = 14.7469 (5) Å
*b* = 14.5697 (4) Å
*c* = 5.8430 (2) Å
β = 92.854 (1)°
*V* = 1253.86 (7) Å^3^
*Z* = 4
Mo *K*α radiation
μ = 0.12 mm^−1^
*T* = 113 K
0.20 × 0.18 × 0.08 mm

#### Data collection

Rigaku Saturn724 CCD diffractometer
Absorption correction: multi-scan (*CrystalClear*; Rigaku/MSC, 2005 ▶) *T*_min_ = 0.976, *T*_max_ = 0.990
12984 measured reflections
3005 independent reflections
2037 reflections with *I* > 2σ(*I*)
*R*_int_ = 0.049

#### Refinement

*R*[*F* ^2^ > 2σ(*F* ^2^)] = 0.036
*wR*(*F* ^2^) = 0.078
*S* = 1.12
3005 reflections
181 parameters
H-atom parameters constrained
Δρ_max_ = 0.22 e Å^−3^
Δρ_min_ = −0.27 e Å^−3^

### 

Data collection: *CrystalClear* (Rigaku/MSC, 2005 ▶); cell refinement: *CrystalClear*; data reduction: *CrystalClear*; program(s) used to solve structure: *SHELXS97* (Sheldrick, 2008 ▶); program(s) used to refine structure: *SHELXL97* (Sheldrick, 2008 ▶); molecular graphics: *SHELXTL* (Sheldrick, 2008 ▶); software used to prepare material for publication: *CrystalStructure* (Rigaku/MSC, 2005 ▶).

## Supplementary Material

Crystal structure: contains datablock(s) global, I. DOI: 10.1107/S1600536812035763/aa2055sup1.cif

Structure factors: contains datablock(s) I. DOI: 10.1107/S1600536812035763/aa2055Isup2.hkl

Supplementary material file. DOI: 10.1107/S1600536812035763/aa2055Isup3.cml

Additional supplementary materials:  crystallographic information; 3D view; checkCIF report

## Figures and Tables

**Fig. 1.**
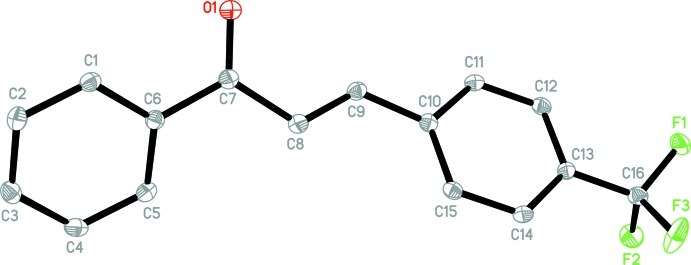
The molecular structure of the title compound. Thermal displacement ellipsoids are drawn at the 30% probability level and H atoms are omitted.

**Fig. 2.**
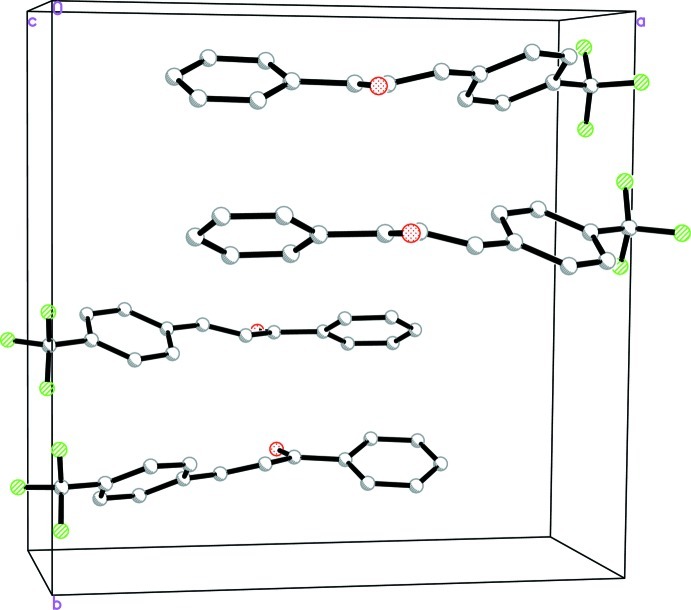
The crystal packing of the title compound.

## supplementary crystallographic information

### Comment

The title compound belongs to the chalcones, which are Michael acceptors and constitute an important group of natural products that belong to the flavonoid family (Dimmock *et al.*, 1999; Go *et al.*, 2005). Natural and synthetic chalcones have been reported to possess strong antiproliferative effects in ovarian cancer cells and in gastic cancer cells (Shibata, 1994; Devincenzo *et al.*, 1995).

The dihedral angle between two benzene rings is 48.8 (2) ° (Fig. 1). The crystal packing shows no π···π or other classical intermolecular interactions (Fig. 2).

### Experimental

In 25 ml round-bottomed flask, the acetophenone (5.0 mmol) and sodium hydroxide (7.5 mmol) were dissolved in ethanol (2 ml), and the mixture was stirred at room temperature for 5 min followed by addition of 4-trifluoromethylbenzaldehyde (5.0 mmol). The reaction mixture was then stirred at room temperature and monitored by TLC until the reaction completed. The solid was filtered, washed with cold water, recrystallized from ethanol, and dried *in vacuo* to give the desired product. Crystals of the title compound were obtained by slow evaporation of the dichloromethane/*n*-hexane solution (1:1) at room temperature. ^1^N NMR(400 MHz, CDCl_3_, TMS): 7.53 (dd, 2H, J = 7.6, 15.6 Hz), 7.60 (m, 2H), 7.68 (d, 2H, J = 8.0 Hz), 7.75 (d, 2H, J = 8.0 Hz), 7.82 (d, 1H, J = 15.6 Hz), 8.03 (d, 2H, J = 8.4 Hz) p.p.m..

### Refinement

All the H atoms were positioned geometrically (C—H = 0.95 Å) and refined as riding with *U*_iso_(H) = 1.2*U*_eq_(C).

### Crystal data

**Table:**

C_16_H_11_F_3_O	*F*(000) = 568
*M**_r_* = 276.25	*D*_x_ = 1.463 Mg m^−^^3^
Monoclinic, *P*2_1_/*c*	Mo *K*α radiation, λ = 0.71073 Å
Hall symbol: -P 2ybc	Cell parameters from 4102 reflections
*a* = 14.7469 (5) Å	θ = 2.0–28.0°
*b* = 14.5697 (4) Å	µ = 0.12 mm^−^^1^
*c* = 5.8430 (2) Å	*T* = 113 K
β = 92.854 (1)°	Prism, colorless
*V* = 1253.86 (7) Å^3^	0.20 × 0.18 × 0.08 mm
*Z* = 4	

### Data collection

**Table:**

Rigaku Saturn724 CCD diffractometer	3005 independent reflections
Radiation source: rotating anode	2037 reflections with *I* > 2σ(*I*)
Multilayer monochromator	*R*_int_ = 0.049
Detector resolution: 14.22 pixels mm^-1^	θ_max_ = 27.9°, θ_min_ = 2.0°
ω and φ scans	*h* = −19→19
Absorption correction: multi-scan (*CrystalClear*; Rigaku/MSC, 2005)	*k* = −14→19
*T*_min_ = 0.976, *T*_max_ = 0.990	*l* = −7→7
12984 measured reflections	

### Refinement

**Table:**

Refinement on *F*^2^	Primary atom site location: structure-invariant direct methods
Least-squares matrix: full	Secondary atom site location: difference Fourier map
*R*[*F*^2^ > 2σ(*F*^2^)] = 0.036	Hydrogen site location: inferred from neighbouring sites
*wR*(*F*^2^) = 0.078	H-atom parameters constrained
*S* = 1.12	*w* = 1/[σ^2^(*F*_o_^2^) + (0.0283*P*)^2^] where *P* = (*F*_o_^2^ + 2*F*_c_^2^)/3
3005 reflections	(Δ/σ)_max_ = 0.001
181 parameters	Δρ_max_ = 0.22 e Å^−^^3^
0 restraints	Δρ_min_ = −0.27 e Å^−^^3^

### Special details

**Table:**

Geometry. All s.u.'s (except the s.u. in the dihedral angle between two l.s. planes) are estimated using the full covariance matrix. The cell s.u.'s are taken into account individually in the estimation of s.u.'s in distances, angles and torsion angles; correlations between s.u.'s in cell parameters are only used when they are defined by crystal symmetry. An approximate (isotropic) treatment of cell s.u.'s is used for estimating s.u.'s involving l.s. planes.
Refinement. Refinement of *F*^2^ against ALL reflections. The weighted *R*-factor *wR* and goodness of fit *S* are based on *F*^2^, conventional *R*-factors *R* are based on *F*, with *F* set to zero for negative *F*^2^. The threshold expression of *F*^2^ > σ(*F*^2^) is used only for calculating *R*-factors(gt) *etc*. and is not relevant to the choice of reflections for refinement. *R*-factors based on *F*^2^ are statistically about twice as large as those based on *F*, and *R*- factors based on ALL data will be even larger.

### Fractional atomic coordinates and isotropic or equivalent isotropic displacement parameters (Å^2^)

**Table:**

	*x*	*y*	*z*	*U*_iso_*/*U*_eq_	
F1	1.01951 (5)	0.12265 (5)	1.04772 (13)	0.0377 (2)	
F2	0.95432 (5)	0.05980 (5)	1.32422 (13)	0.0375 (2)	
F3	0.95565 (5)	0.20580 (5)	1.29461 (15)	0.0493 (3)	
O1	0.47484 (6)	0.13892 (6)	0.27878 (15)	0.0329 (2)	
C1	0.29314 (8)	0.16320 (7)	0.3888 (2)	0.0215 (3)	
H1	0.3095	0.1866	0.2449	0.026*	
C2	0.20299 (9)	0.16081 (7)	0.4417 (2)	0.0243 (3)	
H2	0.1576	0.1834	0.3353	0.029*	
C3	0.17888 (9)	0.12555 (8)	0.6496 (2)	0.0249 (3)	
H3	0.1168	0.1235	0.6851	0.030*	
C4	0.24500 (8)	0.09312 (8)	0.8068 (2)	0.0251 (3)	
H4	0.2280	0.0684	0.9489	0.030*	
C5	0.33571 (8)	0.09691 (8)	0.7565 (2)	0.0225 (3)	
H5	0.3811	0.0761	0.8655	0.027*	
C6	0.36044 (8)	0.13127 (7)	0.5461 (2)	0.0198 (3)	
C7	0.45675 (8)	0.13432 (8)	0.4807 (2)	0.0220 (3)	
C8	0.52985 (8)	0.13323 (8)	0.6661 (2)	0.0230 (3)	
H8	0.5162	0.1474	0.8193	0.028*	
C9	0.61401 (8)	0.11232 (7)	0.6158 (2)	0.0207 (3)	
H9	0.6223	0.0939	0.4623	0.025*	
C10	0.69610 (8)	0.11424 (7)	0.7696 (2)	0.0188 (3)	
C11	0.77584 (8)	0.07768 (7)	0.6888 (2)	0.0202 (3)	
H11	0.7745	0.0490	0.5426	0.024*	
C12	0.85708 (8)	0.08253 (8)	0.8182 (2)	0.0211 (3)	
H12	0.9109	0.0571	0.7615	0.025*	
C13	0.85929 (8)	0.12479 (7)	1.0312 (2)	0.0185 (3)	
C14	0.78038 (8)	0.16112 (7)	1.1158 (2)	0.0202 (3)	
H14	0.7820	0.1899	1.2619	0.024*	
C15	0.69934 (8)	0.15519 (7)	0.9860 (2)	0.0205 (3)	
H15	0.6453	0.1793	1.0449	0.025*	
C16	0.94629 (8)	0.12902 (8)	1.1725 (2)	0.0234 (3)	

### Atomic displacement parameters (Å^2^)

**Table:**

	*U*^11^	*U*^22^	*U*^33^	*U*^12^	*U*^13^	*U*^23^
F1	0.0177 (4)	0.0663 (5)	0.0293 (5)	−0.0017 (4)	0.0027 (3)	0.0043 (4)
F2	0.0314 (5)	0.0496 (5)	0.0311 (5)	0.0037 (3)	−0.0037 (4)	0.0171 (4)
F3	0.0378 (5)	0.0427 (5)	0.0648 (6)	0.0105 (4)	−0.0231 (5)	−0.0281 (4)
O1	0.0250 (5)	0.0531 (6)	0.0208 (5)	−0.0003 (4)	0.0020 (4)	0.0019 (4)
C1	0.0247 (7)	0.0216 (6)	0.0180 (7)	−0.0040 (5)	−0.0002 (5)	0.0000 (5)
C2	0.0216 (7)	0.0227 (7)	0.0282 (8)	−0.0008 (5)	−0.0028 (6)	−0.0013 (5)
C3	0.0217 (7)	0.0240 (7)	0.0291 (7)	−0.0045 (5)	0.0044 (6)	−0.0065 (6)
C4	0.0313 (7)	0.0248 (7)	0.0198 (7)	−0.0074 (6)	0.0059 (6)	−0.0031 (5)
C5	0.0254 (7)	0.0224 (6)	0.0193 (7)	−0.0015 (5)	−0.0023 (5)	0.0004 (5)
C6	0.0206 (6)	0.0190 (6)	0.0198 (7)	−0.0023 (5)	0.0000 (5)	−0.0023 (5)
C7	0.0222 (7)	0.0229 (6)	0.0209 (7)	0.0002 (5)	0.0010 (5)	0.0009 (5)
C8	0.0234 (7)	0.0259 (7)	0.0197 (7)	−0.0017 (5)	0.0008 (5)	−0.0011 (5)
C9	0.0239 (7)	0.0195 (6)	0.0188 (7)	−0.0013 (5)	0.0008 (5)	−0.0010 (5)
C10	0.0206 (6)	0.0157 (6)	0.0201 (7)	−0.0012 (5)	0.0013 (5)	0.0026 (5)
C11	0.0248 (7)	0.0194 (6)	0.0165 (6)	0.0011 (5)	0.0025 (5)	−0.0007 (5)
C12	0.0201 (6)	0.0223 (6)	0.0211 (7)	0.0033 (5)	0.0042 (5)	0.0007 (5)
C13	0.0197 (6)	0.0174 (6)	0.0183 (6)	0.0006 (5)	0.0003 (5)	0.0029 (5)
C14	0.0238 (7)	0.0199 (6)	0.0170 (6)	0.0020 (5)	0.0020 (5)	−0.0013 (5)
C15	0.0199 (7)	0.0208 (6)	0.0211 (7)	0.0031 (5)	0.0048 (5)	0.0005 (5)
C16	0.0224 (7)	0.0255 (7)	0.0226 (7)	0.0032 (5)	0.0027 (5)	−0.0010 (6)

### Geometric parameters (Å, º)

**Table:**

F1—C16	1.3361 (14)	C7—C8	1.4896 (16)
F2—C16	1.3439 (14)	C8—C9	1.3249 (16)
F3—C16	1.3301 (14)	C8—H8	0.9500
O1—C7	1.2244 (14)	C9—C10	1.4715 (16)
C1—C2	1.3802 (17)	C9—H9	0.9500
C1—C6	1.3981 (16)	C10—C11	1.3946 (16)
C1—H1	0.9500	C10—C15	1.3967 (16)
C2—C3	1.3815 (17)	C11—C12	1.3866 (16)
C2—H2	0.9500	C11—H11	0.9500
C3—C4	1.3889 (17)	C12—C13	1.3874 (16)
C3—H3	0.9500	C12—H12	0.9500
C4—C5	1.3850 (16)	C13—C14	1.3914 (15)
C4—H4	0.9500	C13—C16	1.4920 (16)
C5—C6	1.3929 (16)	C14—C15	1.3860 (16)
C5—H5	0.9500	C14—H14	0.9500
C6—C7	1.4897 (16)	C15—H15	0.9500
			
C2—C1—C6	120.29 (12)	C10—C9—H9	116.1
C2—C1—H1	119.9	C11—C10—C15	118.58 (11)
C6—C1—H1	119.9	C11—C10—C9	117.90 (11)
C1—C2—C3	119.95 (12)	C15—C10—C9	123.42 (11)
C1—C2—H2	120.0	C12—C11—C10	121.06 (11)
C3—C2—H2	120.0	C12—C11—H11	119.5
C2—C3—C4	120.34 (12)	C10—C11—H11	119.5
C2—C3—H3	119.8	C11—C12—C13	119.55 (11)
C4—C3—H3	119.8	C11—C12—H12	120.2
C5—C4—C3	119.98 (12)	C13—C12—H12	120.2
C5—C4—H4	120.0	C12—C13—C14	120.32 (11)
C3—C4—H4	120.0	C12—C13—C16	119.73 (11)
C4—C5—C6	119.99 (11)	C14—C13—C16	119.94 (11)
C4—C5—H5	120.0	C15—C14—C13	119.70 (11)
C6—C5—H5	120.0	C15—C14—H14	120.1
C5—C6—C1	119.43 (11)	C13—C14—H14	120.1
C5—C6—C7	122.13 (11)	C14—C15—C10	120.78 (11)
C1—C6—C7	118.44 (11)	C14—C15—H15	119.6
O1—C7—C8	121.12 (11)	C10—C15—H15	119.6
O1—C7—C6	120.32 (11)	F3—C16—F1	106.62 (10)
C8—C7—C6	118.56 (11)	F3—C16—F2	105.91 (10)
C9—C8—C7	119.56 (11)	F1—C16—F2	105.12 (9)
C9—C8—H8	120.2	F3—C16—C13	113.26 (10)
C7—C8—H8	120.2	F1—C16—C13	113.02 (10)
C8—C9—C10	127.71 (12)	F2—C16—C13	112.27 (10)
C8—C9—H9	116.1		
			
C6—C1—C2—C3	−0.92 (17)	C15—C10—C11—C12	−0.73 (17)
C1—C2—C3—C4	0.55 (17)	C9—C10—C11—C12	175.76 (10)
C2—C3—C4—C5	0.65 (17)	C10—C11—C12—C13	−0.31 (17)
C3—C4—C5—C6	−1.48 (17)	C11—C12—C13—C14	0.78 (17)
C4—C5—C6—C1	1.10 (17)	C11—C12—C13—C16	179.16 (10)
C4—C5—C6—C7	−178.18 (10)	C12—C13—C14—C15	−0.18 (17)
C2—C1—C6—C5	0.10 (17)	C16—C13—C14—C15	−178.57 (10)
C2—C1—C6—C7	179.41 (10)	C13—C14—C15—C10	−0.88 (16)
C5—C6—C7—O1	158.69 (12)	C11—C10—C15—C14	1.33 (16)
C1—C6—C7—O1	−20.61 (16)	C9—C10—C15—C14	−174.95 (10)
C5—C6—C7—C8	−22.32 (16)	C12—C13—C16—F3	145.25 (11)
C1—C6—C7—C8	158.38 (10)	C14—C13—C16—F3	−36.36 (16)
O1—C7—C8—C9	−18.27 (17)	C12—C13—C16—F1	23.86 (15)
C6—C7—C8—C9	162.75 (11)	C14—C13—C16—F1	−157.75 (10)
C7—C8—C9—C10	175.00 (10)	C12—C13—C16—F2	−94.84 (13)
C8—C9—C10—C11	171.53 (11)	C14—C13—C16—F2	83.55 (13)
C8—C9—C10—C15	−12.17 (19)		
